# The adaptive traction doughnut resection technique for pyloric cancer: a case of curative resection and stenosis management

**DOI:** 10.1055/a-2739-2527

**Published:** 2025-11-27

**Authors:** Abdeldjalil Sais, Hanae Boutallaka, Jean Grimaldi, Louis-Jean Masgnaux, Tanguy Fenouil, Jérôme Rivory, Mathieu Pioche

**Affiliations:** 1 639305 Department of Gastroenterology and Endoscopy, Groupement Hospitalier Portes de Provence (GHPP), Montélimar, France; 2 26926 Department of Gastroenterology and Digestive Oncology, University Hospital of Saint-Etienne, Saint-Étienne, France; 3 Department of Gastroenterology and Endoscopy, Hôpital Edouard Herriot, Hospices Civils de Lyon, Lyon, France; 4 36609 Department of Digestive Pathology, Hospices Civils de Lyon, Lyon, France

Gastric adenocarcinoma is a leading cause of cancer-related death, but early detection allows for curative endoscopic therapies. While endoscopic submucosal dissection is the standard for early gastric cancer, its application in the pyloric ring is technically demanding due to the narrow lumen, acute angulation, and strong peristalsis, which impede stable access.

We present the case of a 71-year-old man with a 3 cm pyloric lesion confirmed as well-differentiated adenocarcinoma. To overcome the technical difficulties of this location, a “doughnut resection with adaptive traction” was performed ( Video 1 ). This strategy involves a full circumferential incision around the lesion, using retroflexion in the bulb for the initial duodenal side incision followed by a circumferential incision of the gastric edge. An adaptive traction device (ATRACT, Belmont d’azergues, France) was used with one loop attached on the duodenal side and three loops on the gastric edge to facilitate exposure and increase dissection speed. Adaptive traction was done by placing the rubber band in different axes to facilitate exposure while the dissection progressed ( Fig. 1 ).

This video demonstrates the “doughnut resection” technique for a circumferential pyloric adenocarcinoma, showcasing a systematic two-step dissection from both the duodenal and gastric sides to achieve a complete en-bloc resection.Video 1

**Fig. 1:**
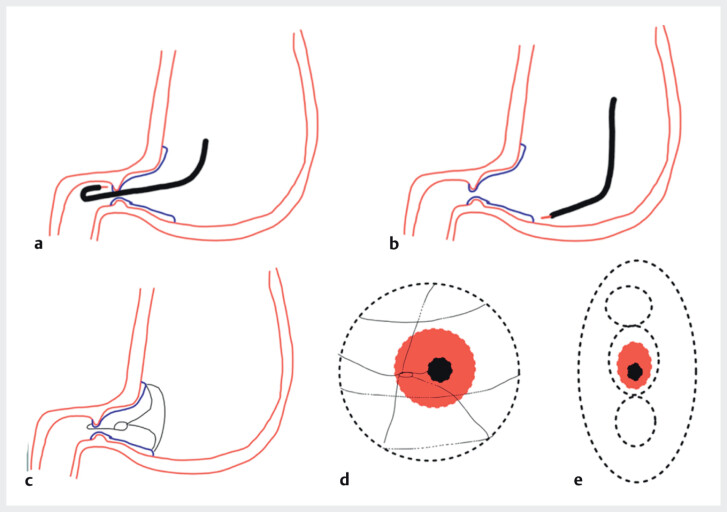
Schematic of the “doughnut resection” technique showing **a** duodenal-side dissection, **b** circumferential gastric-side incision, **c** traction view, **d** traction view, **e** the final view of the traction.

The specimen was retrieved en-bloc, and histopathology confirmed a complete R0 resection ( Fig. 2 ).

**Fig. 2:**
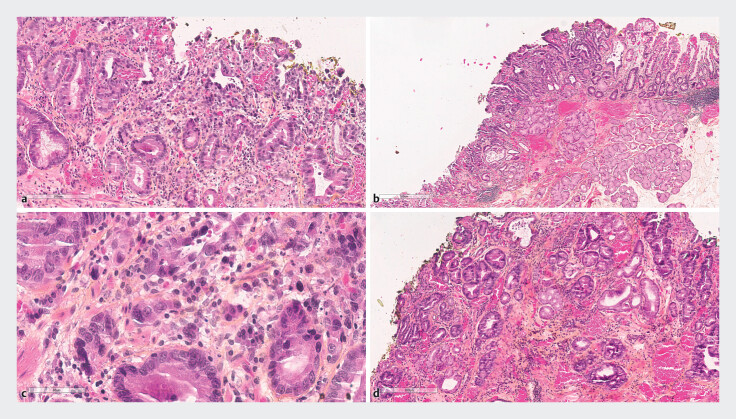
**a** (×5) Microscopic examination revealed an adenocarcinoma of the pyloric region with invasive glands destroying the mucosa but without any invasion in the submucosal space. **b** (×40) The tumoral glands displayed high nuclear pleomorphism but retained a tubular architecture. **c** (×5) The adenocarcinoma extended superficially to the gastro-duodenal junction. **d** (×10) This cancer was developed on chronic and atrophic gastritis with intestinal metaplasia being difficult to proof given the proximity with duodenal mucosa.

One month later, the patient presented with recurrent vomiting and a 7 kg weight loss due to a tight post-operative stenosis. During endoscopy, the stricture was found to be impassable, making conventional balloon dilatation unfeasible. Consequently, a 16 mm fully covered self-expanding metal stent (Hanaro Stent) was placed, leading to the resolution of symptoms. Stent removal was planned after 1 month.

The “adaptive traction doughnut resection” technique is an effective strategy for circumferential pyloric lesions. In the cases of severe subsequent stenosis, primary stenting is a viable alternative when dilatation is not feasible.

Endoscopy_UCTN_Code_TTT_1AO_2AG_3AZ

## Notes

Endoscopy E-Videos https://eref.thieme.de/e-videos
*E-Videos* is an open access online section of the journal *Endoscopy* , reporting on interesting cases and new techniques in gastroenterological endoscopy. All papers include a high-quality video and are published with a Creative Commons CC-BY license. Endoscopy E-Videos qualify for HINARI discounts and waivers and eligibility is automatically checked during the submission process. We grant 100% waivers to articles whose corresponding authors are based in Group A countries and 50% waivers to those who are based in Group B countries as classified by Research4Life (see: https://www.research4life.org/access/eligibility/ ). This section has its own submission website at https://mc.manuscriptcentral.com/e-videos .
